#  Giant Choledochal Cyst in A Neonate

**DOI:** 10.21699/jns.v6i1.408

**Published:** 2017-01-01

**Authors:** Yogesh Kumar Sarin, Prince Raj, Parveen Kumar, Anju Garg

**Affiliations:** Department of Pediaric Surgery, Maulana Azad Medical College, New Delhi, India

**Dear Sir**

Choledochal cysts (CDC) are increasingly diagnosed in antenatal period [1]. Occasionally, these cysts may grow to a very large size and differentiation from other cystic masses of the abdomen can be challenging. There is also a debate regarding the timing of surgery, optimal management strategy, and the type of surgery. Enteric biliary diversion procedures are not only technically difficult but are also associated with the high rates of postoperative complications in the neonatal period [2].

A 20-day-old female child, full term, born by normal vaginal delivery brought to us with complaints of gradually increasing abdominal distention since birth; with intermittent clay colored stools and discoloration of sclera for 10 days. Antenatal scans done at 13 weeks and 32 weeks did not show any abnormality. On examination, jaundice with a large cystic mass, with restricted mobility and involving more than right half of abdominal compartments, was found. Routine investigations were normal ex¬cept liver function test (Total Serum bilirubin 4.8mg%, Direct 2.4mg%) and elevated liver en¬zymes. Ultrasound done elsewhere showed 10.4x7.4cms cystic area noticed around the region of common bile duct (CBD), but due to the large size of the cyst, its origin could not be ascertained. Magnetic resonance cholangiopancreatography (MRCP) (Fig.1) showed a 10.0x9.6x8.4cm thin walled cystic mass lesion in right side abdomen in region of CBD/porta-hepatis with marked dilatation of intra hepatic biliary radicles in both liver lobes. CBD could not be visualized separately and the mass was displacing bowel loops to left. Repeat USG revealed could not improve the diagnosis. Thus, differential diagnoses of giant CDC, Cystic biliary atresia(CBA), and omental cyst with compression of CBD were kept. Patient was taken up for surgery and on exploration a giant choledochal cyst (Fig.2) was found and after aspirating 350 ml of bile the cyst was excised and hapatico-duodenostomy (HD) was done. Postoperative period was uneventful.

**Figure:**
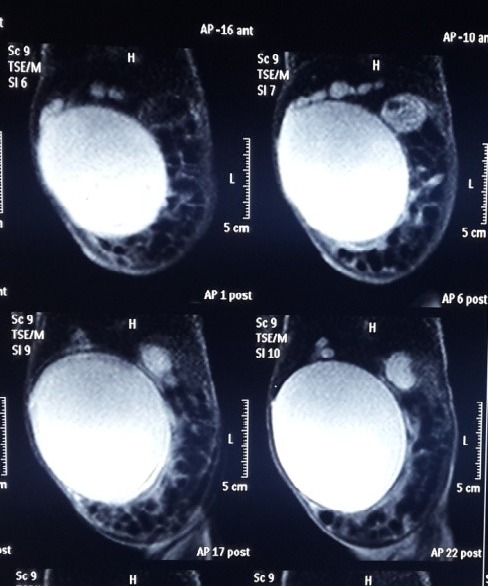
Figure 1: MRCP showing large cyst

**Figure:**
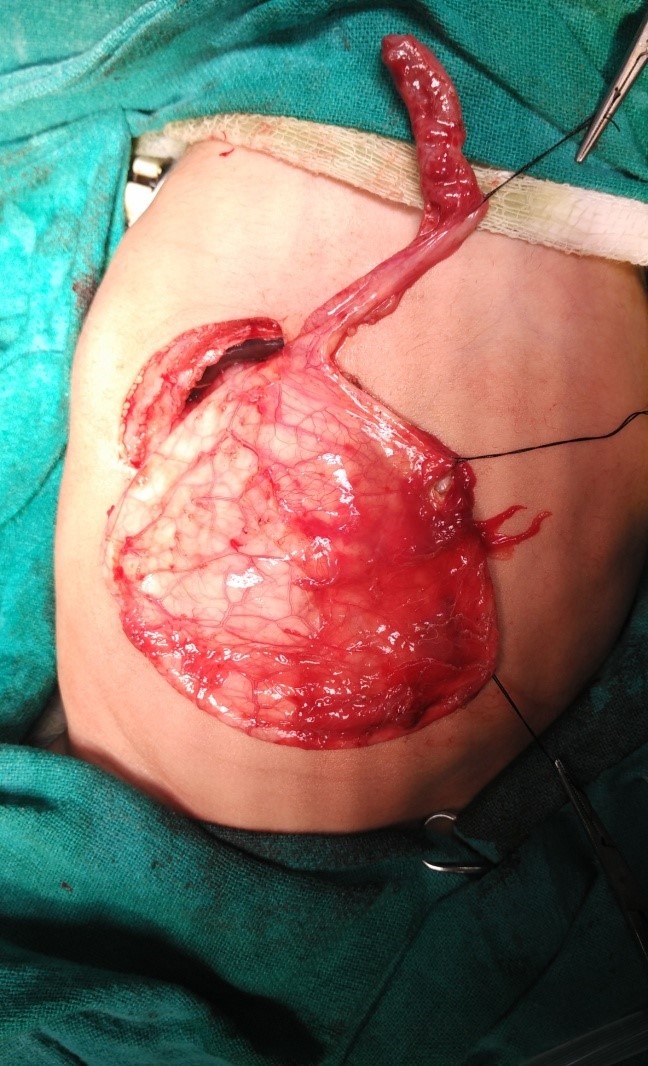
Figure 2: Peroperative view of hugely dilated CDC.

CDC usually presents in the first two decades of life; neonatal presentation is uncommon [4]. The classic triad of abdominal pain, right upper quadrant mass and obstructive jaundice is rare, but it was present in our case. The primary differential of CDC in the neonatal age group is CBA, and it is critical to distinguish these entities, as they have different surgical implications. Delaying surgery in CBA results in poor prognosis. USG plays an important role and is always the first investigation of choice, as most of the times it reliably distinguishes between CDC and CBA. However, USG has its own limitations and evaluating the entire biliary tree is difficult due to limited field of view and poor overview when compared with cross-sectional imaging modalities. MRCP is routinely done and it is considered gold standard as it better delineates the hepatobiliary system and gives an idea about the aberrant vascular and ductal anatomy. It has got high sensitivity (70-100%) and specificity (90-100%) for diagnosing CDC [4]. But sometimes when the cyst is so large that it occupies almost entire abdomen then in that scenario even MRCP fails to reliably pinpoint the organ of origin as it happened in our case.

Excision of cyst and biliary enteric anastomosis is the procedure of choice, and it could be achieved either in the form of Hepatico-duodenostomy (HD) or Roux-en-Y Hepatico-jejunostomy (RYHJ). We preferred HD over RYHJ as it is more physiological, requires less operative time, fewer anastomosis, less chances of leak and less blood loss as compared to RYHJ. Though post of reflux and gastritis are common in HD, we did not find it to be significant. On two-month follow-up, baby has gained weight and thriving well.

## Footnotes

**Source of Support:** Nil

**Conflict of Interest:** None
